# *Mycoplasma hyorhinis*-encoded cytidine deaminase efficiently inactivates cytosine-based anticancer drugs

**DOI:** 10.1016/j.fob.2015.07.007

**Published:** 2015-08-03

**Authors:** Johan Vande Voorde, Peter Vervaeke, Sandra Liekens, Jan Balzarini

**Affiliations:** Rega Institute for Medical Research, KU Leuven, Minderbroedersstraat 10, blok x – bus 1030, B-3000 Leuven, Belgium

**Keywords:** 3TC, 2′,3′-dideoxy-3′-thiacytidine, ara-Cyd, cytosine arabinoside, CDA, cytidine deaminase, (d)Ado, (2′-deoxy)adenosine, (d)Guo, (2′-deoxy)guanosine, (d)Ino, (2′-deoxy)inosine, (d)Urd, (2′-deoxy)uridine, ddC, 2′,3′-dideoxycytidine, dThd, thymidine, dFdC, gemcitabine, dFdU, 2′,2′-difluoro-2′-deoxyuridine, Imm-H, Immucillin-H, NA, nucleoside analogue, PNP, purine nucleoside phosphorylase, Mycoplasma, Cytidine deaminase, Cancer, Nucleoside analogue, Purine nucleoside phosphorylase, Gemcitabine

## Abstract

Mycoplasmas may colonize tumor tissue in patients. The cytostatic activity of gemcitabine was dramatically decreased in *Mycoplasma hyorhinis*-infected tumor cell cultures compared with non-infected tumor cell cultures. This mycoplasma-driven drug deamination could be prevented by exogenous administration of the cytidine deaminase (CDA) inhibitor tetrahydrouridine, but also by the natural nucleosides or by a purine nucleoside phosphorylase inhibitor. The *M. hyorhinis*-encoded CDA_Hyor_ gene was cloned, expressed as a recombinant protein and purified. CDA_Hyor_ was found to be more catalytically active than its human equivalent and efficiently deaminates (inactivates) cytosine-based anticancer drugs. CDA_Hyor_ expression at the tumor site may result in selective drug inactivation and suboptimal therapeutic efficiency.

## Highlights

• Mycoplasmas may colonize tumor tissue in patients.
• Mycoplasma-encoded cytidine deaminase deaminates cytosine-based anticancer drugs.
• The activity of gemcitabine is compromised in mycoplasma-infected tumor cells.
• Gemcitabine activity can be restored by nucleosides or a PNP inhibitor.

## 1 Introduction

Mycoplasmas are considered to be the smallest self-replicating organisms, both in dimension and genome size [1]. They often lack genes that are crucial for different synthetic pathways, including the *de novo* synthesis of purine and pyrimidine bases [2,3]. Therefore mycoplasmas rely on their host tissue from which they scavenge and recycle DNA/RNA precursors using various nucleo(s)(t)ide transporters and salvage enzymes [2,4,5]. Recently, we and others showed that certain catabolic mycoplasma enzymes (i.e. pyrimidine nucleoside phosphorylase, purine nucleoside phosphorylase and cytidine deaminase) interfere with the biological (i.e. cytostatic and antiviral) activity of different therapeutic nucleoside analogues (NAs) by producing less active or inactive drug metabolites. This was demonstrated for both pyrimidine- and purine-derived antimetabolites including gemcitabine, floxuridine, trifluridine, cladribine, and others [6–10]. There have been several reports that mycoplasmas have been shown to preferentially colonize tumor tissue in patients [11–20]. If this phenomenon can be broadly confirmed and since nucleoside-derived drugs are established cornerstones in the chemotherapy of several cancers [21], the presence of such prokaryotes in the tumor microenvironment may be a confounding factor for the efficiency of anticancer nucleoside analogues and of importance for optimization of nucleoside-based cancer treatment [22,23].

**Fig. 1:**
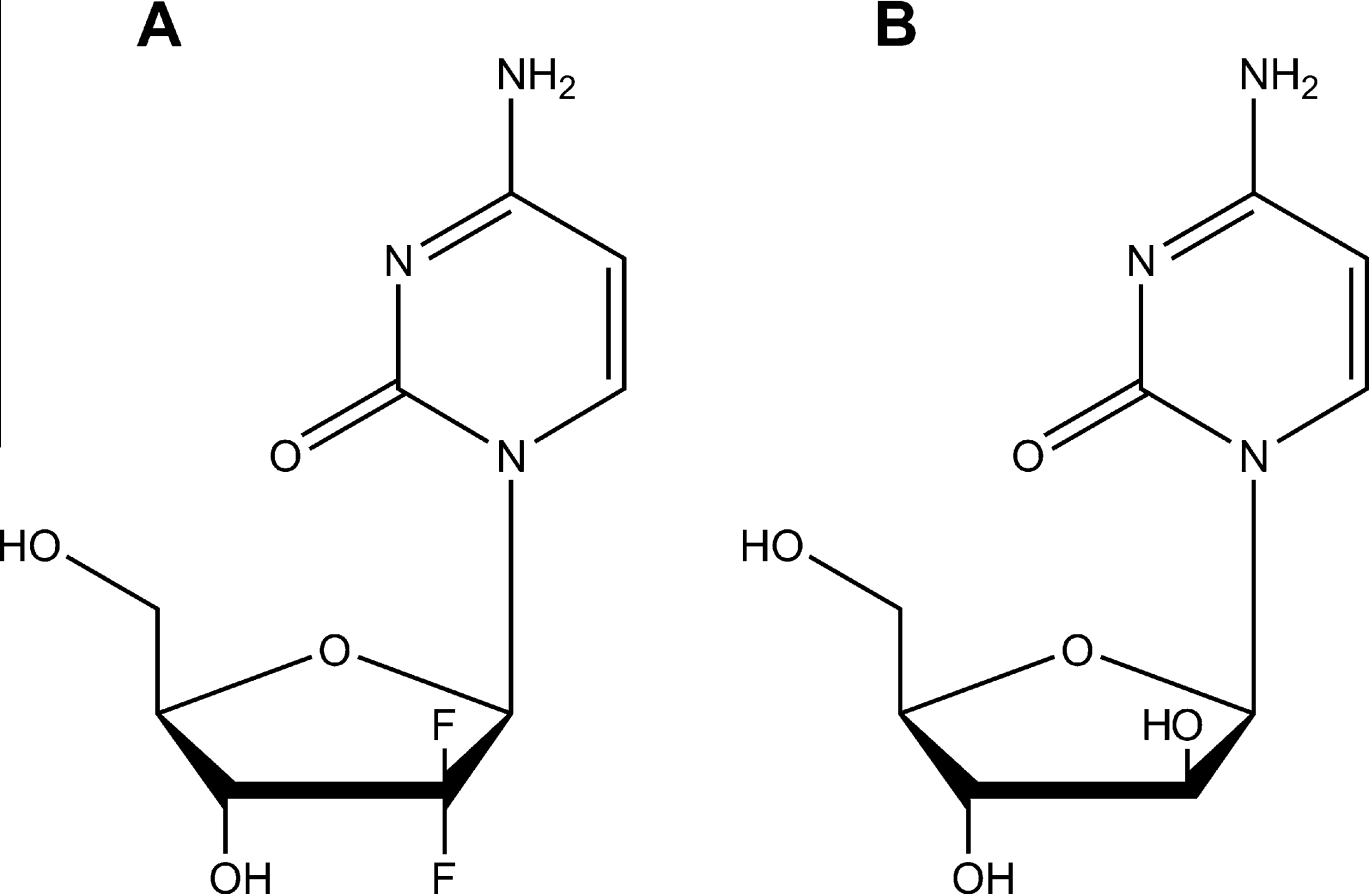
Molecular structure of the (2′-deoxy)cytidine analogues gemcitabine (A) and cytarabine (B).

Recently, we reported efficient CDA-catalyzed deamination of gemcitabine (2′,2′-difluoro-2′-deoxycytidine; dFdC) resulting in a dramatically decreased cytostatic activity (up to 60-fold) of this drug in different *Mycoplasma hyorhinis*-infected tumor cell cultures [10]. Similarly, the response of *M. hyorhinis*-infected tumor xenografts in mice to gemcitabine treatment was significantly lower compared with uninfected control tumors [10]. The biological function of CDA is to catalyze the irreversible deamination of the natural pyrimidine nucleosides cytidine (Cyd) and 2′-deoxycytidine (dCyd) to uridine (Urd) and 2′-deoxyuridine (dUrd), respectively [24]. However, several clinical anticancer (d)Cyd analogues, including gemcitabine and cytarabine (cytosine arabinoside; ara-Cyd) (Fig. 1), can be catabolized by (cellular) drug deamination producing the corresponding, less active, (2′-deoxy)uridine metabolites. These molecules therefore show a decreased cytostatic activity in CDA-overexpressing tumor cells [25,26]. In the present study we biochemically and kinetically characterized *M. hyorhinis*-encoded CDA and report on a surprising interaction between mycoplasma CDA and purine nucleoside phosphorylase (PNP) activity in mycoplasma-infected tumor cells.

## 2 Materials and methods

### 2.1 Chemicals

Nucleosides, nucleoside analogues and inorganic agents were purchased from Sigma–Aldrich (St-Louis, MO) unless stated differently. Gemcitabine (2′,2′-difluoro-2′-deoxycytidine; dFdC) was purchased from Carbosynth (Berkshire, UK). Radioactive [5-^3^H]-gemcitabine ([5-^3^H]-dFdC) (radiospecificity: 12 Ci/mmol) was obtained from Moravek Biochemicals Inc. (Brea, CA). Immucillin-H (Imm-H) was kindly provided by Dr. V. Schramm (Albert Einstein College of Medicine, Bronx, NY).

### 2.2 Cell cultures

Human breast carcinoma MDA-MB-231 cells and *M. hyorhinis* were obtained from the American Tissue Culture Collection (Rockville, MD). Human breast carcinoma MCF-7 cells were kindly provided by Prof. G.J. Peters (Amsterdam, The Netherlands). Cells were infected with *M. hyorhinis* and after two or more passages (to avoid bias by the initial inoculum) successful infection was confirmed using the MycoAlert™ mycoplasma detection kit (Lonza, Basel, Switzerland). Although this assay is only semi-quantitative, a maximal infection was observed three to four days after subculturing the mycoplasma-exposed cells. Chronically *M. hyorhinis*-infected tumor cells are further referred to as MDA-MB-231.Hyor and MCF-7.Hyor. All cells were maintained in Dulbecco’s modified Eagle medium (Invitrogen, Carlsbad, CA) supplemented with 10% foetal bovine serum (Integro, Dieren, The Netherlands), 10 mM HEPES and 1 mM sodium pyruvate (Invitrogen) and grown at 37 °C in a humidified CO_2_-controlled incubator.

### 2.3 Biological assays

The cytostatic activity of dFdC (gemcitabine) was compared in mycoplasma-infected and uninfected tumor cells. MDA-MB-231 and MDA-MB-231.Hyor cells were seeded in 48-well plates (Nunc™, Roskilde, Denmark) at 10,000 cells/well. After 24 h, an equal volume of fresh medium containing gemcitabine [in the presence or absence of natural purine nucleosides (100 μM) or the PNP inhibitor Imm-H (10 μM)] was added. Three days later (to ensure sufficient cell-proliferation and mycoplasma growth), cells were trypsinized and counted in a Coulter counter (Analis, Suarlée, Belgium). The 50% inhibitory concentration (IC_50_) was defined as the compound concentration required to reduce tumor cell proliferation by 50%.

### 2.4 Gemcitabine stability in the supernatant of mycoplasma-infected and uninfected cell cultures

The stability of gemcitabine in spent cell-free but mycoplasma-containing culture medium of confluent MDA-MB-231, MDA-MB-231.Hyor, MCF-7 and MCF-7.Hyor tumor cells was evaluated. Tumor cells were seeded in 75 cm^2^ culture flasks (TTP, Trasadingen, Switzerland). After five days, supernatant was withdrawn and cleared by centrifugation at 300*g* for 6 min to remove (debris of) the tumor cells. Reactions were performed in a final volume of 300 μL containing dFdC (5 μM), [5-^3^H]dFdC (1 μCi), different concentrations of thymidine (dThd), uridine (Urd), adenosine (Ado) or inosine (Ino) and 240 μL spent culture medium. Samples were incubated at 37 °C and after 60 min incubation, 100 μL was withdrawn and ice-cold MeOH was added to a final concentration of 66% MeOH to terminate the enzymatic reactions and to precipitate (remove) macromolecules such as DNA, RNA and proteins. Samples were kept on ice for 10 min and cleared by centrifugation at 16,000*g* for 15 min. The supernatants were withdrawn and analyzed on a reverse phase RP-8 column (Merck, Darmstadt, Germany) using HPLC (Alliance 2690, Waters, Milford, MA). The following gradient (further referred to as gradient A) was used: 10 min linear gradient of 100% buffer A [50 mM NaH_2_PO_4_ (Acros Organics, Geel, Belgium); 5 mM heptane sulfonic acid; pH 3.2] to 98% buffer A + 2% acetonitrile (BioSolve BV, Valkenswaard, the Netherlands); 10 min linear gradient to 90% buffer A + 10% acetonitrile; 5 min linear gradient to 75% buffer A + 25% acetonitrile; 5 min linear gradient to 100% buffer A followed by 10 min equilibration at 100% buffer A. Fractions of 1 mL were collected, transferred to 9 mL OptiPhase HiSafe 3 and radioactivity was counted in a liquid scintillation analyzer.

### 2.5 Purification of *M. hyorhinis* CDA (CDA_Hyor_)

**Fig. 2:**
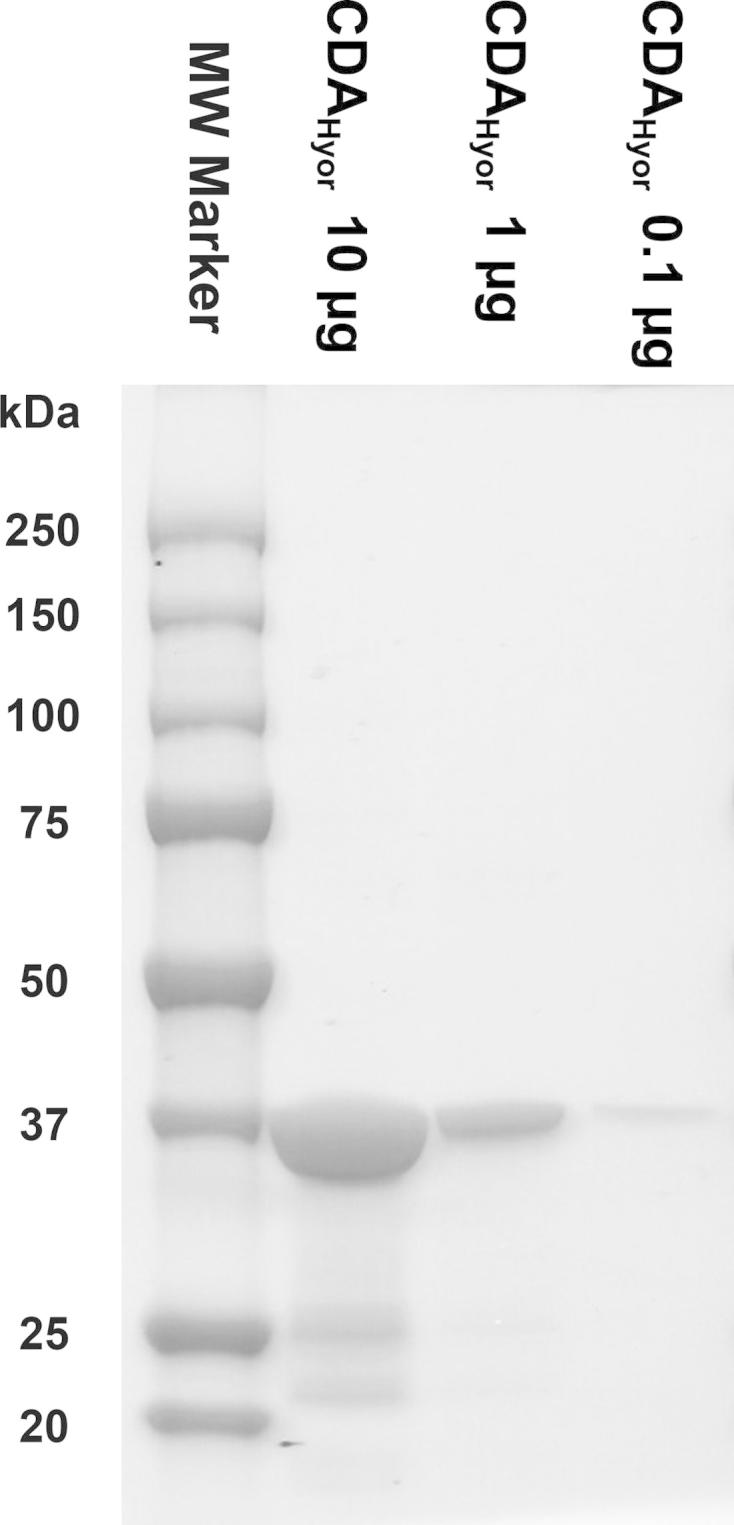
Purity evaluation of the CDA_Hyor_-GST fusion protein. Three different concentrations (i.e. 10, 1 and 0.1 μg) of the purified enzyme preparation were analyzed using SDS–PAGE. Proteins were stained using Bio-Safe™ Coomassie G-250 Stain (Bio-Rad Laboratories, CA, USA).

A codon-optimized DNA sequence encoding the *M. hyorhinis* cytidine deaminase (CDA_Hyor_) was synthetically assembled between the EcoRI and NotI restriction sites of a pIDTsmart vector (Integrated DNA technologies, Coralville, IO). The fragment was subsequently subcloned between the EcoRI and NotI sites of the pGEX-5X-1 bacterial expression vector (Amersham Pharmacia Biotech, Uppsala, Sweden) and CDA_Hyor_ was expressed in *Escherichia coli* as a GST-fusion protein (hereafter referred to as CDA_Hyor_) according to a procedure previously described by Liekens et al. [27]. SDS–PAGE revealed that the protein was of expected size (∼38–40 kDa) and purity (⩾95%) (Fig. 2). Since *E. coli* CDA consists of 294 amino acids, it would be characterized by a molecular weight of around 31 kDa [28]. Therefore, the contaminating protein bands shown in Fig. 2 are not likely related to *E. coli*-encoded CDA.

### 2.6 Enzyme assays

#### 2.6.1 Determination of the substrate specificity of CDA_Hyor_ and CDA_Human_

To study the deamination of different nucleosides and nucleoside analogues by CDA_Hyor_ and CDA_Human_ (ProSpec, Rehovot, Israel) different potential substrates (100 μM) were exposed to both enzymes (80 nM CDA_Hyor_ or 27 nM CDA_Human_) and incubated at 37 °C in PBS in a total volume of 300 μL. At different time points, 100 μL-fractions were withdrawn and the reaction was terminated by heat-inactivation of the enzyme at 95 °C for 3 min. Next, the samples were rapidly cooled on ice for 15 min and cleared by centrifugation at 16,000*g* for 15 min. Nucleosides were separated on a reverse phase RP-8 column (Merck) and quantified by HPLC analysis. For each product UV-based detection was performed at the specific wavelength of optimal absorption.

The separation of (2′-deoxy)cytidine [(d)Cyd] from (2′-deoxy)uridine [(d)Urd] was performed by HPLC using linear gradient B [from 100% buffer A and 2% acetonitrile to 80% buffer A and 20% acetonitrile] as follows: 5 min 100% buffer A; 5 min linear gradient to 80% buffer A + 20% acetonitrile; 5 min linear gradient to 100% buffer A followed by 5 min equilibration at 100% buffer A. Samples containing dFdC, ara-Cyd, 5-aza-2′-deoxycytidine, 5-aza-cytidine, 2′,3′-dideoxycytidine (ddC) or 2′,3′-dideoxy-3′-thiacytidine (3TC) were analyzed by HPLC using linear gradient A as described above.

#### 2.6.2 Kinetic assays

Kinetic studies were performed for the deamination of different substrates [Cyd, dCyd, dFdC and ara-Cyd] by CDA_Hyor_ and CDA_Human_. Deamination was studied at varying substrate concentrations ranging from 100 μM to 45 mM in a reaction containing 4 nM CDA_Hyor_ or 11 nM CDA_Human_ incubated in PBS at 37 °C for 10 min. Samples were processed and analyzed by HPLC as described above. Kinetic parameters (*K*_M_ and *k*_cat_) were determined by means of non-linear regression analysis (using GraphPad Prism5) and the ratio *k*_cat_/*K*_M_ (catalytic efficiency) was calculated.

## 3 Results

### 3.1 Deamination of gemcitabine by mycoplasma CDA can be prevented in the presence of exogenous natural purine and pyrimidine nucleosides

The cytostatic activity of gemcitabine (dFdC) was decreased by ∼36 fold in *M. hyorhinis*-infected MDA-MB-231 breast cancer cell cultures compared with uninfected control tumor cells (Table 1). This could be prevented by co-administration of 250 μM of the cytidine deaminase inhibitor tetrahydrouridine (THU), but also by natural purine nucleosides (i.e. Ado, Ino or Guo) as well as by Immucillin-H (Imm-H), a potent inhibitor of mycoplasma PNP (Table 1).

**Fig. 3:**
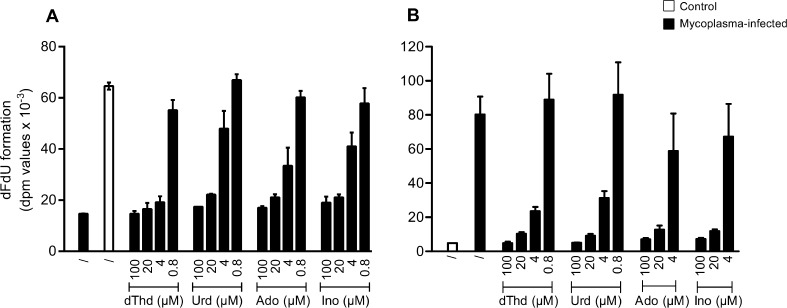
Inhibition of mycoplasma-associated [5-^3^H]dFdU formation by natural nucleosides. Formation of [5-^3^H]dFdU from [5-^3^H]dFdC in the tumor cell-free supernatant of mycoplasma-infected and control MDA.MB.231 (A) and MCF-7 (B) breast cancer cell cultures in the presence/absence of different concentrations of pyrimidine (dThd and Urd) and purine (Ado and Ino) nucleosides. The data are the mean of at least two independent experiments (±S.E.M.).

Since THU could efficiently restore the cytostatic activity of dFdC in the presence of *M. hyorhinis* in the tumor cell culture medium, the stability of radiolabeled dFdC ([5-^3^H]dFdC) was studied in the tumor cell-free (but mycoplasma-containing) culture medium of *M. hyorhinis*-infected MDA-MB-231 (Fig. 3A) and MCF-7 (Fig. 3B) breast cancer cells. A pronounced inhibition of [5-^3^H]-dFdC deamination (i.e. decreased formation of the inactive metabolite [5-^3^H]-dFdU) by 1 mM THU could be observed [10], but also, a dose-dependent inhibition of [5-^3^H]dFdC deamination by the exogenous supply of natural pyrimidine (i.e. dThd and Urd) or purine (i.e. Ado and Ino) nucleosides was observed in the spent tumor cell culture medium (Fig. 3). Also, [5-^3^H]-dFdU formation could be inhibited by exogenous administration of other natural nucleosides such as Guo, dAdo, dIno or dGuo (data not shown).

### 3.2 Substrate specificity of human- and mycoplasma-encoded CDA

The substrate specificity of recombinant *M. hyorhinis* CDA (CDA_Hyor_) was studied and compared with CDA_Human_. Both enzymes catalyzed the deamination of the natural pyrimidine nucleosides Cyd and dCyd, and the well-known anticancer drugs dFdC and ara-Cyd. Deamination of 5-aza-2′-deoxycytidine (decitabine) and 5-aza-cytidine (vidaza), both used for the treatment of myelodysplastic syndromes [29], was also observed in the presence of CDA_Hyor_ but could not be demonstrated for CDA_Human_. The antiviral (i.e. HIV) drugs 2′,3′-dideoxycytidine (ddC; zalcitabine) and 2′,3′-dideoxy-3′-thiacytidine (3TC; lamivudine) were found to be insensitive to deamination by both human and mycoplasma CDA.

### 3.3 Kinetics of human- and mycoplasma-encoded CDA

**Fig. 4:**
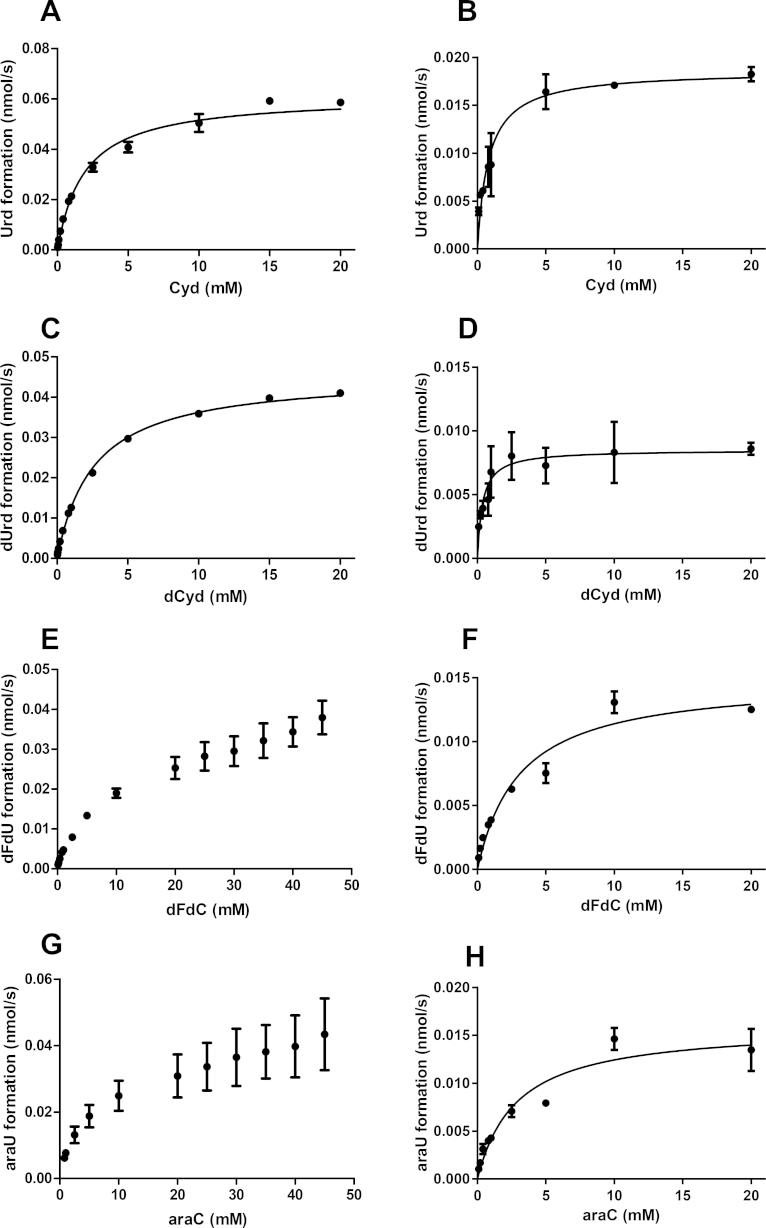
Kinetic analysis of CDA_Hyor_- and CDA_Human_-catalyzed deamination of natural nucleosides and nucleoside analogues Deamination of different concentrations of Cyd (A and B), dCyd (C and D), dFdC (E and F) and ara-Cyd (G and H) by CDA_Hyor_ (A, C, E and G) or CDA_Human_ (B, D, F and H). The data are the mean of at least two independent experiments (±S.E.M.).

The kinetic parameters (*K*_M_ and *k*_cat_) for CDA_Hyor_- and CDA_Human_-catalyzed deamination of Cyd, dCyd, dFdC and ara-Cyd were determined. Relatively high *K*_M_ values (ranging from high micromolar to low millimolar concentrations) were observed for both enzymes. CDA_Hyor_ typically displayed higher *K*_M_ values compared with CDA_Human_ (Tables 2 and 3 and Fig. 4). However, the catalytic efficiency (calculated as *k*_cat_/*K*_M_) of CDA_Hyor_-catalyzed reactions was ∼2–4 fold higher compared with CDA_Human_ (Tables 2 and 3).

Interestingly, deamination of both dFdC and ara-Cyd by CDA_Hyor_, but not by CDA_Human_, was characterized by a second *K*_M_ at very high (and presumably biologically irrelevant) concentrations. However, precise values could not be determined due to insolubility of the highest drug concentrations (∼45 mM in the reaction mixture) tested. The calculated *K*_M_ for dFdC and ara-Cyd as represented in Table 2 was therefore based on the measurements obtained when using up to 30 mM substrate. Measurements obtained using higher concentrations were excluded from the non-linear regression analysis but are still displayed in Fig. 4E (for dFdC) and Fig. 4G (for ara-Cyd). For these above-mentioned reasons, we preferred not to draw a curve line fitting the experimental data points in Fig. 4E and G.

## 4 Discussion

Recent studies have focused on the role of commensal prokaryotes in the efficiency of chemotherapeutic cancer treatment. For example, an intact intestinal microbiome seems to be essential for optimal response to immune therapy and platinum-, doxorubicin- or cyclophosphamide-based cancer chemotherapy [30,31]. Conversely, the presence of some prokaryotes (e.g. mycoplasmas) in the tumor microenvironment may negatively influence the outcome of nucleoside-based treatment of cancer [23]. A thorough understanding of the implications of the (tumor) microbiome on cancer chemotherapy may ultimately lead to a more optimal treatment strategy (e.g. by combination therapy with antibiotics or specific prokaryotic enzyme inhibitors) and may also attenuate adverse side-effects.

In this report, we investigated the catabolic action of mycoplasma-encoded cytidine deaminase against the anticancer nucleoside analogues gemcitabine and cytarabine. To the best of our knowledge, this study is the first to describe the biochemical properties of the CDA encoded by *M. hyorhinis*, a commensal that has been repeatedly reported to preferentially associate with tumor tissue in patients. We found that CDA_Hyor_ does not only catalyze the deamination of natural cytidine and 2′-deoxycytidine but also of several clinical nucleoside antimetabolites, including gemcitabine and cytarabine. The efficiency of substrate conversion catalyzed by CDA_Hyor_ was consistently higher compared with the human equivalent enzyme. In addition, we observed deamination of 5-aza-2′-deoxycytidine and 5-aza-cytidine by CDA_Hyor_ but not by CDA_Human_ under similar experimental conditions. Based on these observations it could be expected that pronounced inactivation of different anticancer (2′deoxy)cytidine analogues may occur in mycoplasma-infected tumors leading to reduced efficacy.

Indeed, expression of CDA_Hyor_ explains the predominantly high levels of (the poorly cytostatic) [5-^3^H]dFdU derived from radiolabeled gemcitabine in the culture medium of mycoplasma-infected tumor cell cultures and, as a result, the consequently decreased cytostatic activity of this drug in these tumor cell cultures (Table 1) and xenografts in mice [10]. Somewhat surprisingly, we found that the stability and biological (cytostatic) activity of gemcitabine in mycoplasma-infected tumor cell cultures could be restored by (i) the co-administration of natural pyrimidine (i.e. dThd and Urd) and purine (i.e. Ado, Ino and Guo) nucleosides and (ii) administration of a specific PNP inhibitor. Interestingly, inhibition of *Escherichia coli* and human CDA by different nucleosides [i.e. dThd, (d)Urd, (d)Ado and (d)Guo] has been reported earlier [32–34]. Previously, we have shown that *M. hyorhinis* PNP is responsible for the catabolism of different purine nucleoside analogues, including cladribine and fludarabine [9]. It can be hypothesized that *M. hyorhinis-*related PNP activity may also indirectly contribute to the deamination of cytidine analogues by depletion of those intracellular purine nucleosides that seem to act as natural inhibitors of CDA_Hyor_. Exogenous administration of these natural nucleosides may then restore the depleted purine nucleoside pools and, as a result, also the cytostatic potential of gemcitabine. This hypothesis would then also explain the rescuing cytostatic activity of gemcitabine by Immucillin-H, a potent and selective PNP inhibitor. Earlier, we reported a similar concerted action between CDA_Hyor_ and the *M. hyorhinis*-encoded pyrimidine nucleoside phosphorylase which catabolizes dThd, Urd and dUrd [10]. However, when exposing purified CDA_Hyor_ to different substrates (Cyd, dCyd or dFdC) in the presence of natural purine nucleosides or dThd, we could not observe decreased deamination (data not shown). It is therefore unlikely that the observed rescue of gemcitabine is due to a direct interaction of CDA_Hyor_ with purine nucleosides or dThd. Although inhibition of CDA by (d)Urd may be explained by end-product feedback inhibition, the mechanism of CDA_Hyor_-inhibition by dThd and the purine nucleosides remains unclear. In this respect, it cannot be excluded that interaction (i.e. inhibition) of CDA can be attributed to the mono-, di- or triphosphorylated derivatives of the natural nucleosides that were found inhibitory to CDA_Hyor_-catalyzed deamination of gemcitabine. Alternatively, the natural nucleosides may compete for uptake with dFdC by *M. hyorhinis* and therefore lower the amount of drug to be deaminated intracellularly by the mycoplasma.

In conclusion, we have kinetically characterized the *M. hyorhinis*-encoded CDA and found it more catalytically efficient than human CDA. It deaminates (inactivates) anticancer drugs such as gemcitabine and cytarabine, but also 5-aza-(2′-deoxy)cytidine. Its deaminating action may negatively affect the cytostatic activity of anti-cancer drugs such as gemcitabine, but could be annihilated by co-administration of natural nucleosides or a specific PNP inhibitor.

## Figures and Tables

**Table 1:** Cytostatic activity of gemcitabine (dFdC) in MDA-MB-231 and MDA-MB-231. Hyor cells in the absence/presence of the selective CDA inhibitor tetrahydrouridine, natural purine nucleosides or the selective purine nucleoside phosphorylase inhibitor Immucillin-H (Imm-H). Results are the mean ± S.D. of at least two independent experiments.

	IC_50_^a^ value of dFdC (μM)					
	As such	+THU (250 μM)	+Ado (100 μM)	+Ino (100 μM)	+Guo (100 μM)	+Imm-H (10 μM)
MDA-MB-231	0.0042 ± 0.00041	0.004 ± 0.0003	0.0031 ± 0.0014	0.0037 ± 0.00076	0.0042 ± 0.0012	0.0037 ± 0.00076
MDA-MB-231.Hyor	0.15 ± 0.016	0.004 ± 0.0001	0.0096 ± 0.0049	0.0099 ± 0.0042	0.025 ± 0.0089	0.0051 ± 0.0021

Fold difference	∼36	1	∼3	∼3	∼6	∼1.5

a 50% Inhibitory concentration or compound concentration required to inhibit tumor cell proliferation by 50%.

**Table 2:** Kinetic parameters of CDA_Hyor_. The *K*_M_ and *k*_cat_ values ± S.E.M. for the natural substrates of CDA_Hyor_ and for gemcitabine and cytarabine were determined using nonlinear regression analysis (using GraphPad Prism 5) from data obtained in at least two independent experiments.

	*K*_M_ (μM)	*k*_cat_ (s^−1^)	*k*_cat_/*K*_M_ (s μM)^−1^
Cytidine	1898 ± 163	171 ± 5	0.090
2′-Deoxycytidine	2586 ± 114	127 ± 2	0.049
dFdC	9064 ± 1487	105 ± 7	0.012
ara-Cyd	6172 ± 2336	119 ± 13	0.019

**Table 3:** Kinetic parameters of CDA_Human_. The *K*_M_ and *k*_cat_ values ± S.E.M. for the natural substrates of CDA_Human_ and for gemcitabine and cytarabine were determined using nonlinear regression analysis (using GraphPad Prism 5) from data obtained in at least two independent experiments.

	*K*_M_ (μM)	*k*_cat_ (s^−1^)	*k*_cat_/*K*_M_ (s μM)^−1^
Cytidine	811 ± 181	17 ± 1	0.021
2′-Deoxycytidine	373 ± 138	7.8 ± 0.64	0.021
dFdC	3080 ± 598	14 ± 0.91	0.004
ara-Cyd	2853 ± 741	15 ± 1.3	0.005

## References

[1] Razin S., Yogev D., Naot Y. (1998). Molecular biology and pathogenicity of mycoplasmas. Microbiol. Mol. Biol. Rev..

[2] Finch L.R., Mitchell A., Maniloff J., McElhaney R.N., Finch L.R., Baseman J.B. (1992). Sources of nucleotides.

[3] Pollack J.D., Williams M.V., McElhaney R.N. (1997). The comparative metabolism of the mollicutes (Mycoplasmas): the utility for taxonomic classification and the relationship of putative gene annotation and phylogeny to enzymatic function in the smallest free-living cells. Crit. Rev. Microbiol..

[4] Neale G.A., Mitchell A., Finch L.R. (1984). Uptake and utilization of deoxynucleoside 5′-monophosphates by *Mycoplasma mycoides* subsp. *mycoides*. J. Bacteriol..

[5] Tham T.N., Ferris S., Kovacic R., Montagnier L., Blanchard A. (1993). Identification of *Mycoplasma pirum* genes involved in the salvage pathways for nucleosides. J. Bacteriol..

[6] Bronckaers A., Balzarini J., Liekens S. (2008). The cytostatic activity of pyrimidine nucleosides is strongly modulated by *Mycoplasma hyorhinis* infection: implications for cancer therapy. Biochem. Pharmacol..

[7] Jetté L., Bissoon-Haqqani S., Le Francois B., Maroun J.A., Birnboim H.C. (2008). Resistance of colorectal cancer cells to 5-FUdR and 5-FU caused by *Mycoplasma* infection. Anticancer Res..

[8] Vande Voorde J., Gago F., Vrancken K., Liekens S., Balzarini J. (2012). Characterization of pyrimidine nucleoside phosphorylase of *Mycoplasma hyorhinis*: implications for the clinical efficacy of nucleoside analogues. Biochem. J..

[9] Vande Voorde J., Liekens S., Balzarini J. (2013). *Mycoplasma hyorhinis*-encoded purine nucleoside phosphorylase: kinetic properties and its effect on the cytostatic potential of purine-based anticancer drugs. Mol. Pharmacol..

[10] Vande Voorde J., Sabuncuoglu S., Noppen S., Hofer A., Ranjbarian F., Fieuws S., Balzarini J., Liekens S. (2014). Nucleoside-catabolizing enzymes in mycoplasma-infected tumour cell cultures compromise the cytostatic activity of the anticancer drug gemcitabine. J. Biol. Chem..

[11] Chan P.J., Seraj I.M., Kalugdan T.H., King A. (1996). Prevalence of mycoplasma conserved DNA in malignant ovarian cancer detected using sensitive PCR-ELISA. Gynecol. Oncol..

[12] Kidder M., Chan P.J., Seraj I.M., Patton W.C., King A. (1998). Assessment of archived paraffin-embedded cervical condyloma tissues for mycoplasma-conserved DNA using sensitive PCR-ELISA. Gynecol. Oncol..

[13] Huang S., Li J.Y., Wu J., Meng L., Shou C.C. (2001). Mycoplasma infections and different human carcinomas. World J. Gastroenterol..

[14] Pehlivan M., Itirli G., Onay H., Bulut H., Koyuncuoglu M., Pehlivan S. (2004). Does *Mycoplasma* sp. play role in small cell lung cancer?. Lung Cancer.

[15] Pehlivan M., Pehlivan S., Onay H., Koyuncuoglu M., Kirkali Z. (2005). Can mycoplasma-mediated oncogenesis be responsible for formation of conventional renal cell carcinoma?. Urology.

[16] Yang H., Qu L., Ma H., Chen L., Liu W., Liu C., Meng L., Wu J., Shou C. (2010). *Mycoplasma hyorhinis* infection in gastric carcinoma and its effects on the malignant phenotypes of gastric cancer cells. BMC Gastroenterol..

[17] Apostolou P., Tsantsaridou A., Papasotiriou I., Toloudi M., Chatziioannou M., Giamouzi G. (2011). Bacterial and fungal microflora in surgically removed lung cancer samples. J. Cardiothorac. Surg..

[18] Urbanek C., Goodison S., Chang M., Porvasnik S., Sakamoto N., Li C.Z., Boehlein S.K., Rosser C.J. (2011). Detection of antibodies directed at *M. hyorhinis* p37 in the serum of men with newly diagnosed prostate cancer. BMC Cancer.

[19] Barykova Y.A., Logunov D.Y., Shmarov M.M., Vinarov A.Z., Fiev D.N., Vinarova N.A., Rakovskaya I.V., Baker P.S., Shyshynova I., Stephenson A.J., Klein E.A., Naroditsky B.S., Gintsburg A.L., Gudkov A.V. (2011). Association of *Mycoplasma hominis* infection with prostate cancer. Oncotarget.

[20] Erturhan S.M., Bayrak O., Pehlivan S., Ozgul H., Seckiner I., Sever T., Karakok M. (2013). Can mycoplasma contribute to formation of prostate cancer?. Int. Urol. Nephrol..

[21] Jordheim L.P., Durantel D., Zoulim F., Dumontet C. (2013). Advances in the development of nucleoside and nucleotide analogues for cancer and viral diseases. Nat. Rev. Drug Discov..

[22] Liekens S., Bronckaers A., Balzarini J. (2009). Improvement of purine and pyrimidine antimetabolite-based anticancer treatment by selective suppression of mycoplasma-encoded catabolic enzymes. Lancet Oncol..

[23] Vande Voorde J., Balzarini J., Liekens S. (2014). An emerging understanding of the Janus face of the human microbiome: enhancement versus impairment of cancer therapy. J. Antimicrob. Chemother..

[24] Vincenzetti S., Cambi A., Neuhard J., Garattini E., Vita A. (1996). Recombinant human cytidine deaminase: expression, purification, and characterization. Protein Expr. Purif..

[25] Neff T., Blau C.A. (1996). Forced expression of cytidine deaminase confers resistance to cytosine arabinoside and gemcitabine. Exp. Hematol..

[26] Yoshida T., Endo Y., Obata T., Kosugi Y., Sakamoto K., Sasaki T. (2010). Influence of cytidine deaminase on antitumour activity of 2′-deoxycytidine analogs *in vitro* and *in vivo*. Drug Metab. Dispos..

[27] Liekens S., Bilsen F., De Clercq E., Priego E.M., Camarasa M.J., Perez-Perez M.J., Balzarini J. (2002). Anti-angiogenic activity of a novel multi-substrate analogue inhibitor of thymidine phosphorylase. FEBS Lett..

[28] Carlow D.C., Carter C.W., Mejlhede N., Neuhard J., Wolfenden R. (1999). Cytidine deaminases from *B. subtilis* and *E. coli*: compensating effects of changing zinc coordination and quaternary structure. Biochemistry.

[29] Parker W.B. (2009). Enzymology of purine and pyrimidine antimetabolites used in the treatment of cancer. Chem. Rev..

[30] Viaud S., Saccheri F., Mignot G., Yamazaki T., Daillere R., Hannani D., Enot D.P., Pfirschke C., Engblom C., Pittet M.J., Schlitzer A., Ginhoux F., Apetoh L., Chachaty E., Woerther P.L., Eberl G., Berard M., Ecobichon C., Clermont D., Bizet C., Gaboriau-Routhiau V., Cerf-Bensussan N., Opolon P., Yessaad N., Vivier E., Ryffel B., Elson C.O., Dore J., Kroemer G., Lepage P., Boneca I.G., Ghiringhelli F., Zitvogel L. (2013). The intestinal microbiota modulates the anticancer immune effects of cyclophosphamide. Science.

[31] Iida N., Dzutsev A., Stewart C.A., Smith L., Bouladoux N., Weingarten R.A., Molina D.A., Salcedo R., Back T., Cramer S., Dai R.M., Kiu H., Cardone M., Naik S., Patri A.K., Wang E., Marincola F.M., Frank K.M., Belkaid Y., Trinchieri G., Goldszmid R.S. (2013). Commensal bacteria control cancer response to therapy by modulating the tumour microenvironment. Science.

[32] Hosono H., Kuno S. (1973). The purification and properties of cytidine deaminase from *Escherichia coli*. J. Biochem..

[33] Vita A., Amici A., Cacciamani T., Lanciotti M., Magni G. (1985). Cytidine deaminase from *Escherichia coli* B. Purification and enzymatic and molecular properties. Biochemistry.

[34] Cacciamani T., Vita A., Cristalli G., Vincenzetti S., Natalini P., Ruggieri S., Amici A., Magni G. (1991). Purification of human cytidine deaminase: molecular and enzymatic characterization and inhibition by synthetic pyrimidine analogs. Arch. Biochem. Biophys..

